# From Tucson to Genomics and Transgenics: The Vector Biology Network and the Emergence of Modern Vector Biology

**DOI:** 10.1371/journal.pntd.0000343

**Published:** 2009-03-31

**Authors:** Barry J. Beaty, Denis J. Prager, Anthony A. James, Marcelo Jacobs-Lorena, Louis H. Miller, John H. Law, Frank H. Collins, Fotis C. Kafatos

**Affiliations:** 1 Department of Microbiology, Immunology, and Pathology, Colorado State University, Fort Collins, Colorado, United States of America; 2 Strategic Consulting Services, Clyde Park, Montana, United States of America; 3 Department of Microbiology & Molecular Genetics, University of California Irvine, Irvine, California, United States of America; 4 Department of Molecular Biology & Biochemistry, University of California Irvine, Irvine, California, United States of America; 5 Department of Molecular Microbiology and Immunology, Malaria Research Institute, Johns Hopkins School of Public Health, Baltimore, Maryland, United States of America; 6 Malaria Vaccine Development Branch, National Institutes of Allergy and Infectious Diseases, National Institutes of Health, Rockville, Maryland, United States of America; 7 Department of Biochemistry and Molecular Biophysics, University of Arizona Tucson, Tucson, Arizona, United States of America; 8 Department of Entomology, University of Arizona Tucson, Tucson, Arizona, United States of America; 9 Department of Entomology, University of Georgia, Athens, Georgia, United States of America; 10 Department of Biological Sciences, University of Notre Dame, Notre Dame, Indiana, United States of America; 11 Faculty of Natural Sciences, Imperial College London, London, United Kingdom; Yale University School of Medicine, United States of America

## Introduction

The research enterprise is often charged with responding to emerging scientific needs and opportunities involving important public health issues. The establishment of the Vector Biology Network (VBN) by The MacArthur Foundation to address critical scientific, research, and human resource needs in vector biology is a model of how to respond to such a charge efficiently and effectively. When the Network was formed in 1989, the resurgence of vector-borne diseases, including malaria and dengue, had revealed critical research and training needs in vector biology. Major knowledge gaps in vector molecular biology and pathogen transmission limited development of new approaches for vector and disease control. The loss of vector biologists/medical entomologists complicated control of these diseases. Members of the VBN, an international consortium of research laboratories, collaborated in the development and application of modern molecular and genetic approaches in vector biology and in the training of a new cadre of scientists capable of developing and applying modern tools to combat these diseases. The VBN exploited the expertise and resources of the consortium institutions, and exceeded all of its milestones, including: development of molecular and genomic approaches in vector biology; genetic transformation of insect vectors; characterization of vector immune systems; identification of molecular and biological bases of vector competence; and exploitation of identified vulnerabilities to interfere with pathogen transmission. The VBN recruited many established scientists from other fields and trained a new generation of leaders in vector biology. Most importantly, the VBN participated in and helped catalyze a remarkable renaissance in vector biology/medical entomology.

## A Case Study of Collaborative Development of a Research Field

Research on mosquitoes and other arthropod vectors of infectious diseases is of great importance for tackling neglected tropical diseases in the developing world. Vector biology is now a well-established and rapidly advancing field that is providing new understanding of vectors and vector pathogen interactions and creating new opportunities for vector-borne disease (VBD) control. Many key milestones and recruitment and training of new researchers in this field were made possible by the coalescence of several laboratories in the US and Europe into a collaborative research network focused on the biology of disease vectors. The Vector Biology Network (VBN) was established and funded for ten years (1990–2000) by The John D. and Catherine T. MacArthur Foundation. The VBN was reinforced through endorsement and, in some cases, coordinated funding by the World Health Organization (WHO) and national research agencies and foundations, which collectively contributed to a remarkable renaissance of vector biology. The history of the VBN provides a valuable case study of how effective strategies can be developed and deployed to address cost-effectively newly recognized scientific challenges related to important public health and social goals.

## The Challenge: Resurgence of Vector-Borne Diseases

The stimulus for developing the VBN was the worldwide resurgence of VBDs. While extraordinary advances in antibiotics and vaccines have controlled many infectious diseases, VBDs continue to afflict hundreds of millions of humans annually, resulting in inestimable morbidity and misery and millions of deaths in disease-endemic countries [1]. These diseases are resurging in areas where they were previously controlled and are newly emerging in other locations. VBD resurgence is associated with multiple causes, including: *biological factors* such as the lack of efficacious vaccines and therapeutics, development of drug resistance in pathogens and pesticide resistance in arthropod vectors, and limitations on pesticide usage because of safety and environmental concerns; *infrastructural factors* such as the deterioration of public health systems for VBD surveillance and control, and attrition of scientists and public health practitioners trained in medical entomology or tropical medicine; and *demographic and social factors* such as rapid population growth, rampant unplanned urbanization in the tropics, and human migration into undeveloped areas containing new pathogens and reservoir hosts for known pathogens.

Because of these factors, the prognosis for the control of VBDs was bleak. New targets and approaches for control were critical, and major knowledge gaps in vector biology needed to be addressed in order to increase the armamentarium for disease control.

## Opportunities and Roadblocks: The Need To Infuse Genetics, Molecular Biology, and Technology into Vector Biology

By the early 1980s, major conceptual and technological advances in molecular biology and genetics were reshaping biomedical research. The manipulation of DNA with recombinant techniques revolutionized the study of gene expression, development, evolution, and population biology. Genetic transformation of *Drosophila* emerged as a powerful approach to gene identification and characterization in a model arthropod [2]. Rapid progress in the development of inexpensive, widely applicable techniques for gene sequencing fostered genome-wide rather than gene-by-gene approaches in biomedicine. This explosion of molecular technology and knowledge created unparalleled opportunities for rapid advances even in neglected fields; indeed, new molecular studies of pathogens revealed exciting opportunities for development of diagnostics, drugs, and vaccines. However, the study of insect vectors of human disease progressed more slowly for several reasons: (i) the relatively small number of scientists working in the area; (ii) the strong focus of medical entomologists on fieldwork rather than on basic laboratory research; (iii) the historical dissociation in American academia of medical entomology (in schools of agriculture) and parasitology (in schools of medicine), which hindered integration of knowledge on the interaction of parasites with both their human and insect hosts; and (iv) the assumption that new more effective anti-parasite drugs and vaccines were imminent.

The small size and difficulty of laboratory maintenance of many vectors complicated their study through existing approaches and tools. For good reasons, most insect scientists focused on the biochemistry and physiology of more tractable non-vector model species. The genetics of *D. melanogaster* and the physical size of moths (especially *Bombyx*, *Cecropia*, and *Manduca*) made them attractive model systems. In contrast, vectors were inconvenient to maintain, genetic information was lacking, and biochemical approaches were daunting; therefore, vector species were largely neglected. This resulted in major knowledge gaps, and vectors were depicted as “black boxes” in natural pathogen transmission cycles. Vector competence and vectorial capacity were operationally rather than mechanistically defined. There was a clear need for the application of modern molecular technology for illuminating the black box of the vector and for training a new generation of vector biologists well-versed in modern technology.

Early applications of simple molecular tools to VBDs allowed identification of cryptic vector species, detection of insecticide resistance, and facilitated the analysis of disease epidemiology and the targeting of interventions. The remarkable taxonomic and population analyses of African mosquitoes of the *Anopheles gambiae* complex by Mario Coluzzi and colleagues [3] were pioneering applications to vectors of tools developed for chromosome mapping in *Drosophila*. Molecular tools contributed to the elucidation of species complexes [4], the genetics and biochemistry of insecticide resistance [5], and basic functions such as reproduction and vitellogenesis in mosquitoes [6]. These early studies provided a powerful paradigm that encouraged development of molecular and genetic tools for vectors. The VBN was formed to explore such tools toward identifying new targets and approaches for surveillance, prevention, and control of vectors and VBDs.

## Roots of The MacArthur Foundation Vector Biology Network

The John D. and Catherine T. MacArthur Foundation was established in the late 1970s. Reflecting their personal interests, perspectives on societal needs, and the Foundation's location in Chicago, the Foundation's new Board of Directors selected mental health and urban issues for emphasis, and established the MacArthur Fellows Program to recognize and reward extraordinary creative potential in individuals.

When Dr. Jonas Salk joined the Board in the early 1980s, he began a series of meetings with health experts to identify areas of special need and opportunity for the new Foundation. A momentous meeting was held with Dr. Kenneth Warren, then the Director of the Rockefeller Foundation's Program on the Great Neglected Diseases of Mankind, an international network of research units collaborating on the diseases of poor countries with a focus on malaria. Dr. Warren, confident that the time was right for a major effort to understand these diseases at the molecular level, convinced Dr. Salk that this was a significant opportunity for a new foundation. Dr. Salk, in turn, persuaded the Foundation's Board.

### Parasite Biology Consortium

After a year of preparation by Foundation staff, The MacArthur Foundation established in 1984 a five-year program of research and supporting activities to reduce the global burden of illness due to parasitic diseases. This commitment was based on the conviction that application of modern molecular and cellular biology, genetics, and immunology would greatly accelerate progress in understanding and combating these diseases. The Foundation decided to pursue this ambitious goal through a relatively novel approach. Rather than supporting individual researchers through separate, discrete grants, it funded an international group of laboratories—the Consortium on the Biology of Parasitic Diseases (Parasite Biology Consortium)—committed to collaborating on applying the latest biological techniques to research on the molecular biology of parasites. (The Parasite Biology Consortium Laboratories were at Case Western Reserve University, Columbia University, Harvard University, Johns Hopkins University, National Polytechnic Institute (Mexico City), New York University, Stanford University, University of California Berkeley, Walter and Eliza Hall Institute (Melbourne, Australia), the Weizmann Institute of Science (Revohot, Israel), and Yale University.) The Foundation also supported efforts that would complement the Consortium, including: the WHO Special Programme for Research and Training in Tropical Diseases (WHO-TDR); the Marine Biological Laboratory's Summer Course on Molecular Parasite Biology at Woods Hole; a program of small workshops and conferences; and discretionary and technical support grants to further the work of the Consortium and advance the field of parasite biology.

### The Formation of the Vector Biology Consortium

When the Parasite Biology Consortium was established, vector biology was even further behind than parasite biology in the application of modern techniques. Three Consortium laboratories (based at Case Western Reserve, Harvard, and Yale Universities) had research groups working on the biology of parasite vectors, but, at least initially, the importance of such studies was not recognized by the Foundation. However, by the mid 1980s, it was becoming evident that the effective control of parasitic diseases in tropical endemic areas would require a multifaceted strategy aimed at both preventing and treating parasitic infections and controlling their transmission by insects. Moreover, there was a growing sense at the Foundation that vector biology was a field ripe for rejuvenation based on the introduction of the molecular and genetic methods that were revolutionizing the study of parasites.

This perspective led the MacArthur Foundation in 1985 to convene a small, one-day meeting of scientists to explore how the Foundation might promote this development. Participants included: B. Beaty (Colorado State University (CSU)), M. Coluzzi (University of Rome), F. Kafatos (Harvard University, and IMBB, Crete), T. Mahowald (Case Western Reserve University (CWRU)), L. Miller (National Institutes of Health (NIH)), L. Riddiford (University of Washington), and D. Prager, the Foundation Program Officer. The consensus of the meeting participants was that the time was right for a major research effort directed toward: understanding, at the molecular and genetic levels, the properties of insect vectors that foster parasite infection and transmission; and developing mechanisms for interfering with these processes.

## Establishment of and Mandate for the Vector Biology Network

Over the next two years, the ideas from that meeting germinated, and led the Foundation to create a new research network focused on vector molecular biology to complement the Parasite Biology Consortium. The Vector Biology Network would include vector laboratories within the Parasite Biology Consortium, and would also exploit newly emerged opportunities. These included the commitment of F. Kafatos to devote 50% of his laboratory to vector biology; the opportunity for A. James, then at Harvard, to start a new vector research program at the University of California Irvine; the convergence of pertinent expertise, infrastructure, and capacity at the NSF-funded Center for Insect Sciences at the University of Arizona; and the establishment of the Arthropod-borne and Infectious Diseases Laboratory at CSU. The VBN laboratories and respective program leaders (PLs) were CWRU (A. Mahowald and M. Jacobs-Lorena), Yale (R. Tesh), Harvard and Crete (F. Kafatos), CSU (B. Beaty), University of Arizona (J. Hildebrand), University of California Irvine (A. James), and NIH (L. Miller). A VBN consortium laboratory was subsequently established at the Centers for Disease Control–Division of Parasitic Diseases, which later moved to the University of Notre Dame (F. Collins).

The VBN was proposed formally to the MacArthur Foundation Board at the time of the renewal of the Parasite Biology Consortium (December 1989) and received unanimous approval. The Foundation specified a clear mandate: 1) establishing and legitimizing a credible field of parasite vector molecular biology with strong intellectual and methodological foundations; 2) attracting to the field established biologists familiar with the latest advances in molecular and cellular biology and genetics; 3) training future generations of parasite vector biologists.

### Development of Specific Goals and Strategy: The First Meeting of the Program Leaders (PLs)

This critical meeting of the VBN PLs was held at The MacArthur Foundation in Chicago on November 20–21, 1989. The intent of the meeting was to reach consensus on long-range goals, initial priorities, strategies for mobilizing intellectual, scientific, and technical resources; and on defining major activities and responsibilities of the PLs. It was agreed that progress in understanding and controlling disease transmission by insect vectors would require an understanding of the relationship between molecular and behavioral events, and close integration of the new discipline into the larger fields of tropical medicine, medical entomology, epidemiology, and ecology. The challenge was to bridge the whole spectrum of inquiry from the molecular to the ecological. The overarching goal for the VBN was to infuse modern molecular biological and genetic techniques into vector biology.

Research priorities and goals for the VBN that emerged from this meeting included: a) development and exploitation of genomic approaches in vector biology; b) development of techniques for genetic transformation of insect vectors; c) characterization of the immune system of vectors; d) determination of the molecular and biological bases of vector competence and vectorial capacity; e) exploitation of identified vulnerabilities to develop effective strategies for interfering with pathogen transmission.

An equally critical outcome of this meeting was the emergence, from the beginning, of an interactive and cohesive VBN spirit based on shared vision, goals, and philosophy. This rapport led to a coherent and systematic plan for accelerating the development of a new field, vector molecular biology. The adopted strategic elements included: a) regular PL meetings, two to three per year, plus broader Network meetings; b) collaborative research programs targeting major scientific goals; c) involvement of established scientists from other fields conversant with modern molecular biological and genetic techniques; d) training both within Network laboratories and in a formal course; e) an efficient administrative structure to facilitate achievement of Network goals.

The collaborative research approach was critical to the success of the Network. It exploited the separate and combined expertise of the Network laboratories in a way that maximized flexibility in achieving Network goals.

### Network Meetings: Sustaining the Network and the Field

The collaborative spirit of the first meeting of the PLs was continued in the subsequent meetings and diffused into the Network as a whole. The ethos was sharing, before publication, of all data, research achievements, experimental models, biological materials, and draft manuscripts; use of research facilities; and visits or exchanges among Network laboratories. In addition, strategic meetings and shared training programs were designed to tackle roadblocks and address opportunities for moving the field forward to stimulate vector biology research, and to accelerate application of new information on the control of parasite transmission in tropical endemic areas.

The PL and other meetings were critical for solidifying the program's cohesion and effective operation. Small (30–40 participants) and intensive (2–3 day) **workshops** proved to be an efficient mechanism for developing effective strategies to overcome specific technical barriers to progress in the field. They included network and non-network experts with pertinent experience in the topic of interest, and were designed to maximize interaction and discussion; each one addressed a specific topic: genofmics, transformation, or bioinformatics. Network-wide **institutes** were devoted principally to VBN participants with one or two plenary speakers from outside the Network; they focused on VBN scientific issues and promoted networking and collaboration among Network scientists.

### Engaging the Broader Community: Key Partnerships

Key to the success of the new enterprise was inclusiveness: the early and sustained involvement of other leaders and proponents of vector biology from multiple institutions and agencies. Particularly noteworthy were joint planning and coordinated funding activities with: (i) the National Institutes of Health (NIH) through Drs. S. James and K. Aultman (Program Officers for vector biology and related fields at the National Institute of Allergy and Infectious Diseases [NIAID]); (ii) WHO-TDR through two successive Directors, Drs. T. Godal and C. Morel, and Dr. B. Dobrokhotov, manager of the Biology and Control of Vectors program at TDR; and (iii) foundations such as the Wellcome Trust and the Howard Hughes Medical Institute. These collaborations played a large role in promoting the field and developing the personnel and tools needed to address the problems.

### The Tucson Meeting and Its Impact

A major goal of the VBN was the genetic manipulation of vectors to block their ability to transmit pathogens. A seminal meeting entitled “Prospects for Malaria Control by Genetic Manipulation of its Vectors” was held January 27–31, 1991, in Tucson, Arizona, and was sponsored by The MacArthur Foundation, WHO-TDR, and the University of Arizona. Participants included scientists with expertise in basic molecular biology, genetics, epidemiology, entomology, vector control, and public health (Figure S1). By the end of the meeting, a consensus had emerged that the use of molecular approaches to vector and disease control should be pursued as a real possibility and not as an impossible dream. The official report of the meeting was published by WHO-TDR (TDR/BCV/MAL-ENT/91). On this basis, TDR established a 20-year plan for the development of malaria refractory mosquitoes. The first projects of the plan focused on genome mapping, identification of selectable markers, and transposable elements, and on database construction.

The TDR's 20-year plan was accelerated as DNA methods became increasingly powerful and less expensive, and manipulating and sequencing mosquito genomes became feasible.

The ability to coordinate research efforts through the VBN resulted in the achievement of a key milestone of the TDR plan—transgenic mosquitoes. A PL meeting at the Biology of Disease Vectors (BDV) course in Crete in 1994 resulted in a VBN plan to develop transgenesis as a tool in vector biology. Transformation of the vector of dengue fever, *Aedes aegypti*, was published in 1998 by the group of A. James, in collaboration with F. Collins [7],[8], and of the malaria vectors *An. stephensi* [9] by the teams of A. Crisanti, F. Kafatos, and C. Savakis, and *An. gambiae* [10] by M. Benedict and colleagues.

VBN workshops on genomics and vector parasite interactions led to the mapping of pathogen refractory genes, and a VBN institute led to the initiation of genomics and informational Web sites for *An. gambiae* and *Ae. aegypti*. Finally, initial discussions between L. Miller, F. Collins, and F. Kafatos at the BDV course in Bamako, Mali (1997), led to draft VBN plans for fully sequencing *Anopheles gambiae* and to the first *Anopheles* Genome Meeting, which was organized by TDR and the VBN in Geneva [11] to discuss potential strategies. Independent sequencing plans were developed by Genoscope and P. Brey at Institut Pasteur. The first *An. gambiae* expressed-sequence tag (EST) sequencing project was co-funded in 2000 by the VBN, NIH-NIAID, and WHO-TDR. An *An. gambiae* Genome Summit was convened in March 2001 in Paris by TDR and Institut Pasteur. One and a half years later, the full genome sequence was completed at Celera Genomics and at Genoscope, with funding from NIAID and the French Ministry of Research, respectively, and was authored by the *An. gambiae* Consortium [12].

## Vector Biology Network Funding, Activities, and Impact

The VBN was supported by relatively modest funding from The MacArthur Foundation (USD 1.2 million total annually for eight laboratories over ten years) to catalyze and promote the field of molecular vector biology. Yet the impact on vector biology was extraordinary. Metrics of success in this goal include: recruitment of established scientists into the field, research productivity of VBN and its collaborators, training of new vector biologists, vector biology publications in high-quality journals, external funding (largely by NIH-NIAID), and vector biology presentations and sessions at the Annual Meeting of The American Society of Tropical Medicine and Hygiene (ASTMH).

### Recruitment of New Scientists into the Field of Vector Molecular Biology

The Network recruited and eventually co-opted outstanding, nationally and internationally recognized scientists of other specialties into the field of vector biology. In many cases, the respective VBN teams were gradually transformed from a focus on Lepidoptera and *Drosophila* research or virology research into leading vector research laboratories. Numerous young scientists were recruited into the field as postdoctoral fellows or junior faculty associated with the network research laboratories and/or as participants in the BDV course.

### VBN Research Accomplishments

Remarkable research accomplishments were made by the laboratories of the VBN and their collaborators, consistent with the research priorities and goals set by the Network. Beyond the milestones of culicine and anopheline transformation and genomic analysis, major advances in vector gene manipulation, molecular characterization, and functional analysis resulted from research activities of the VBN groups, their collaborators, and many other laboratories. These included: the use of microsatellites to establish a genetic map (e.g., [13]); BAC cloning and in situ hybridization of chromosomes; cloning microdissected chromosome regions (e.g., [14]); use of molecular markers to characterize vector chromosomes and genomes, and to identify cryptic species [15]–[19]; development and use of robust tools and approaches to follow gene flow within and between species (e.g., [20]–[26]); exciting new information concerning the molecular biology of vector immunity (e.g., [27]), olfaction and host-seeking (e.g., [28]), salivary gland biology (e.g., [29],[30]), and blood meal digestion and interactions with pathogens (e.g., [31]–[36]); introduction of virus-based transducing systems for gene expression and characterization in vectors (e.g., [37]–[39]); and the proof of concept that molecular intervention can lead to the disruption of pathogen transmission by vectors [40]–[42]. These goals were attained in just over ten years and, together with important achievements of other colleagues in this expanding field, firmly reestablished vector biology as a dynamic field.

The characterization of the mosquito immune system [27] is an excellent example of how VBN effort supported the development of a vibrant new field. A VBN WHO-supported pilot EST/cDNA cloning and sequencing project [43] accelerated the process, and VBN scientists leveraged resources and initiated new collaborations in vector immunity. Comparative biology approaches and transfer of information from *D. melanogaster* allowed innate immunity studies in *An. gambiae* to progress well, even prior to genome sequencing. With the publication of the *An. gambiae* genome, research in vector immunity exploded for both anopheline and culicine mosquitoes (e.g., [44]–[46]). The new tools and information developed during these exciting years led directly to landmark demonstrations by VBN laboratories of: the role of insect innate immunity in vector competence (e.g., [47],[48]), new robust RNA interference methods for gene function analysis in vectors (e.g., [47]), the RNAi response as a robust immune response to arboviruses in vectors (e.g., [49]), and the molecular manipulation of vectors to make them resistant to dengue and malaria transmission (e.g., [50],[51]). The recent publication of the *Ae. aegypti* genome [52] and multiple other vector genome projects in progress undoubtedly will contribute to the productivity of the field. The establishment by the NIH of VectorBase [53] to coordinate and promulgate molecular information on vectors is testimony to the explosion of information, knowledge, tools, laboratories, and investigators in vector biology.

### VBN Training

Training was an integral part of the VBN strategic plan from the beginning. Much of the training of postdoctoral fellows, graduate students, and faculty occurred in the respective VBN research laboratories. Important additional training activities included Network development of the BDV course, publication of corresponding textbooks, and VBN faculty participation in WHO-TDR regional training activities in population genetics and bioinformatics.

Development of the BDV course was one of the first decisions of the Network PLs and was inaugurated in 1990. The VBN members at Colorado Sate University hosted the first several years, and in 1994 it rotated to overseas venues to broaden participation and facilitate the inclusion of scientists and students from disease-endemic countries. Host countries included: Greece (Institute of Molecular Biology and Biotechnology in Crete), Mali (Faculty of Medicine, Pharmacy and Odonto-Stomatology), Brazil (Foundation at the Institute of Oswaldo Cruz), Czech Republic (University of Southern Bohemia and Institute of Parasitology), Mexico (Institute Nacional de Salud Publica), Thailand (Mahidol University), and the United Kingdom (Liverpool School of Tropical Medicine).

This intensive two-week course, a total immersion in vector biology, provided a common background and conceptual framework for a new generation of researchers, who could apply modern approaches to the study and control of disease vectors. It also was invaluable for networking among students, and between students and faculty experts. The course was aimed both at scientists newly recruited into the field from diverse areas and for those with more conventional training in VBDs. Advanced graduate students, postdoctoral fellows, and independent investigators were introduced to the biology of disease vectors, with emphasis on current molecular approaches. A mini-symposium was held each year and emphasized a topic of importance to vector biology. These topics included the biology of vector–parasite interactions, *Ae. aegypti* and *An. gambiae* biology and control, pesticide resistance and stewardship, advances in vector molecular biology and tools, and bioinformatics and genomics. Each year, approximately 35 students and 25 faculty participated in the course. Faculty members were scientists from the VBN, other universities, government institutions, and biotechnology companies from around the world. Every year 16 or more nations were represented. Small class size and the selection of renowned scientists for faculty provided an unparalleled learning and networking experience. Admission was very competitive. To date, 558 students from 67 countries and 154 faculty from 21 countries have participated in the course. The course was considered by WHO-TDR to be one of its flagship training programs.

The first course was restricted to VBN laboratories as a means of testing the curriculum and of promoting interaction among Network participants. All of the PLs, key research personnel, and postdoctoral fellows were in attendance, creating a coherent and unified learning framework that promoted Network evolution and maturation. This contributed to a common vision for the expanding Network, and resulted in a true community of scientists working toward common goals. Subsequent courses were sponsored principally by The MacArthur Foundation and WHO-TDR, but also received support from the Howard Hughes Medical Institute and the NIH, permitting financial assistance to students and faculty from developing countries. Many students and faculty described the course as a career- and life-changing experience. Furthermore, students from the early years have emerged as leaders in the field and as faculty in the course. Most of the new vector biology positions in academia and government in the US in recent years have been filled by former BDV course participants.

After the course had been offered for several years, it became clear that the syllabus provided the basis for a textbook on vector biology with emphasis on modern molecular and quantitative approaches. The textbook *The Biology of Disease Vectors* [54] was published by the University Press of Colorado in 1996 and was provided free of charge to students and faculty in the course and to institutions in disease-endemic countries. An updated edition of *The Biology of Disease Vectors* [55] was published by Elsevier in 2005.

### Network Participation in Building a Scientific Community

Network members became key contributors in the development of vector biology as a field. Network members: (i) organized WHO-TDR workshops throughout the world on vector population genetics and molecular taxonomy, vector bioinformatics, and vector control; (ii) were central to the emergence of vector genome projects; (iii) contributed to the development of the Keystone Symposium “Toward the Genetic Modification of Arthropods,” which became an important meeting for vector and arthropod biologists; (iv) established the “Molecular and Population Biology of Mosquitoes and Other Vectors” workshop held biannually in Crete, which has become a key meeting for vector biologists; (v) organized sessions in ASTMH meetings, Gordon Conferences, and specialty meetings; (vi) participated in the BDV courses; and (vii) trained innumerable visiting scientists, postdoctoral fellows, and students in new tools and techniques in their respective laboratories.

### The Renaissance of Vector Biology

**Figure 1:**
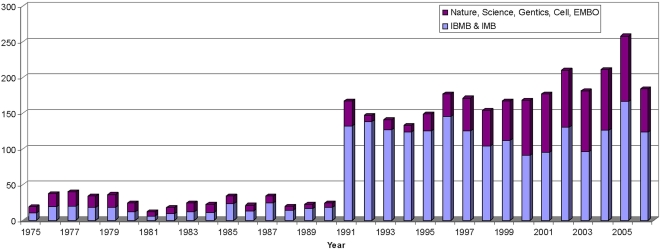
Vector molecular biology publications in leading scientific journals from 1975 to 2006. The number of publications was determined using a Web of Science database search for publications involving mosquitoes, ticks, sand flies, and other vectors. These two groups of leading journals were selected: generalist (*PNAS*, *Science*, *Nature*, *Cell*, *Genetics*), and field-specific (*Insect Molecular Biology*, *Insect Biochemistry & Molecular Biology*).

**Figure 2:**
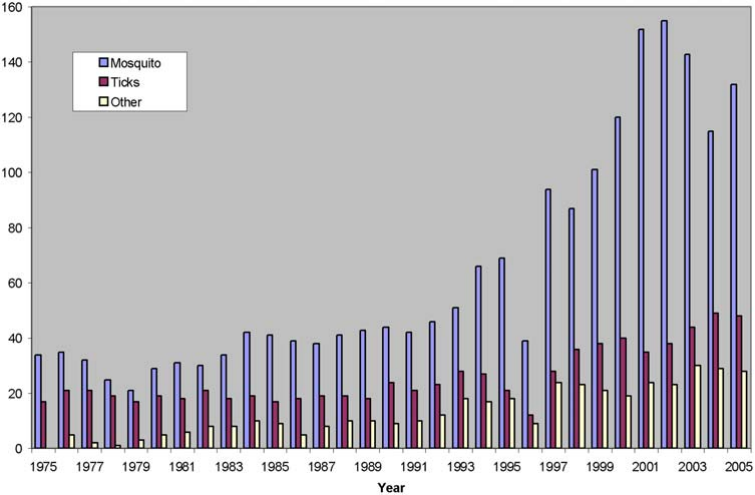
Vector biology grants funded by the National Institutes of Health from 1975 to 2005. The CRISP database was used to search for grants related to mosquitoes, ticks, and other vectors (e.g., sand flies, reduviids, tsetse, etc.).

**Figure 3:**
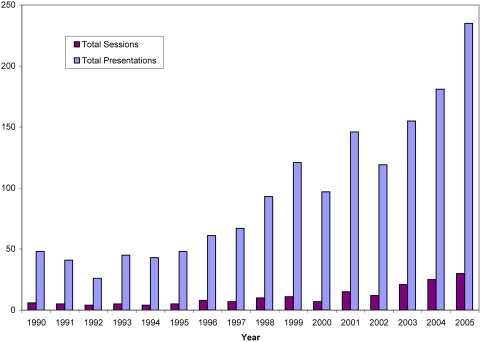
Vector biology presentations and posters at the annual meeting of the American Society of Tropical Medicine and Hygiene from 1990 to 2005. Sessions include symposia, scientific sessions, and poster sessions specifically for vectors. Presentations include oral presentations or posters within vector sessions. The results do not include presentations or sessions organized for specific diseases even if the disease is vector-borne.

The overarching goal of the VBN was to catalyze the development of the vector biology field in the era of molecular biology. A number of specific metrics of success are evident following analysis of time periods before and after the formation of the VBN. An impressive and sustained surge occurred in vector biology publications in leading journals (Figure 1). Prior to 1990, typically fewer than 20 vector biology papers appeared in these journals, but a tenfold increase in the number of publications began in 1991, and has now reached 150 to 200 publications per year in these journals alone. This increase reflects the recruitment of new scientists to the field, the quality of their science, and the development of new tools and approaches to investigate vectors. Concomitant with the increase in publications, a substantial increase is observed in the number of vector biology grants at the US National Institutes of Health (Figure 2), principally the NIAID. Most of this increase is in mosquito-related grants, reflecting in part the influence of the *An. gambiae* genome project. The portfolio of vector biology grants grew from approximately 50 to >120 in the years of the VBN (1990–2000). While the overall NIH-NIAID budget doubled during this period, increases in vector-related studies outpaced this increase. Furthermore, the portfolio continued to increase during the reporting period, reflecting the firm establishment of the new discipline. This latter achievement is emphasized with the formation of a new dedicated NIH study section in Vector Biology. The NIH intramural program also increased, with vector biology research equalling malaria research for the first time. The final indicator of the development of vector biology is the number of scientific presentations and sessions at the annual meeting of a relevant and representative scientific society, The American Society of Tropical Medicine and Hygiene. Review of the annual meeting programs from 1990 to 2005 show that vector biology sessions and presentations increased over the years, from typically fewer than 50 in the early 1990s to more than 200 in 2005 (Figure 3). The addition of new journals with emphases in vector biology, e.g., *Insect Molecular Biology*, *Vector-Borne and Zoonotic Diseases*, *Journal of Insect Sciences*, also is testimony to the resurgence of vector biology.

The Network laboratories contributed to a surge in these metrics but, importantly, the increases reflect the emergence of a widespread, robust new field involving many institutions, programs, and principal investigators in both developed and disease-endemic countries. Although the limited funding for the VBN inevitably disappointed some early practitioners who could not be included in the program, ultimately everyone benefited from the robust development of the field and its funding.

## Lessons Learned—The Funder's Perspective

Collaborative research networks were a relatively new phenomenon when the VBN was established in 1989, and represented an experiment in organizing science to tackle some of society's most significant challenges. For that reason, it is helpful to reflect on the work of the VBN, on how it was structured, and on how it operated. The following thirteen areas represent key lessons from the VBN's experience.

### Network Concept

The research network is a complement to, not a replacement for, individual research supported by traditional funding sources. It is designed to add value to individual scientific pursuits by providing a setting in which the talents of individuals can be mobilized in a reinforced manner to address scientific issues of common concern: by conducting a kind of research that would not otherwise be possible, and by bringing diverse research strategies to bear on overcoming conceptual and technical barriers to progress. Networks can play a particularly powerful and influential role in providing the core leadership, legitimacy, and momentum needed to establish a new scientific field.

### Timing

If the time is right and the environment ready, launching an initiative such as the VBN is like throwing a match on dry tinder; if it is not, even the most creative strategy may fail to spark a fire. The time was right for creating the Network, in terms of recognized need and opportunity; committed people; availability of methods, technologies, and facilities from other fields; and cooperative institutions.

### Receptivity

Critical to the success of a new collaborative venture is the receptivity of the field to such an intervention. Many VBN members are from entomology and *Drosophila* genetics fields in which collaboration is a part of the culture. Months before VBN members received grants awarded by the Foundation, they began collaborating.

### People

Collaboration and communication take place between and among individuals, not institutions. The most critical elements in the success of a collaborative research endeavor are the professional and personal attributes of the participants, and the degree to which they meld into a collegial, open, and productive collective. Effective collaborators are those who: (i) have a demonstrated commitment to interdisciplinary collaboration as a strategy for overcoming substantive and technical barriers to progress in the field; (ii) are collegial, with excellent group process skills, and a demonstrated capacity to work with others; (iii) possess personal and professional maturity and security, an openness to new ideas and approaches, and an ability to reach beyond current paradigms and strategies; and (iv) are willing to make the requisite commitment of time and energy to listen, learn, and make collaboration work.

### Commitment to Shared Goals

The members of the VBN were, to a person, focused intensely on doing whatever it took to advance the field of parasite vector biology and, through their work together, to strengthen the potential of that field to produce strategies for controlling parasitic diseases by interrupting their transmission by insect vectors.

### Face-to-Face Interactions

The Network PLs met, on average, three times a year. These meetings were like lab meetings in which the PLs: (i) trusted each other and freely presented the results of work under way in their laboratories; (ii) discussed barriers to progress and how to overcome them, collectively; and (iii) developed plans for advancing the work of the VBN and strengthening the field of molecular vector biology. These meetings fostered important personal and professional relationships that continue today, as well as a sense of shared purpose and coherent direction.

### Training

Training was integral to the VBN, not an add-on. Each participating laboratory trained several students and fellows, almost all of whom have gone on to establish their own laboratories focused on vector biology. Working in laboratories characterized by high-quality science, open communication, and state-of-the-art facilities and technologies, trainees were immersed in the collaborative ethos that was the VBN's hallmark. Beyond their primary laboratory, trainees benefited from visits to other Network laboratories, participation in the Biology of Disease Vectors Course, attendance at the Network institutes and workshops, and presentations at professional conferences and symposia.

### Biology of Disease Vector Course

The annual BDV course played a critical role in building the field of molecular vector biology around the world. In addition, it served as an anchor for the Network: VBN PLs designed the Course and constituted the core faculty; a meeting of the PLs was held during the Course each year; all VBN trainees took the Course early in their training experience; and the Course brought VBN scientists and trainees into contact with many of the current and future leaders in the field from around the world.

### Workshops

Network-sponsored workshops mobilized communal expertise and experience to tackle the critical barriers to progress in the field. External experts who had grappled with similar challenges in other fields were invited to share their experiences and to advise the Network on how best to proceed. As a result, these sessions were instrumental in the development of strategies that were successful in overcoming barriers and moving the field forward.

### Flexibility

Within the boundaries of expectation established by the Foundation, VBN laboratories were given very wide latitude in how they expended the grant funds provided. As a result, Network laboratories were able to spend the money in a timely and optimal mode, directed at the Network's goals and without constraints of required funder approval for trying novel approaches (for which scientists were the best judges).

### Invisible Administration

An effective research network is one in which administration and logistics are effective, efficient, seamless, and invisible. Toward this end, each Network laboratory had an individual whose job it was to handle the administrative aspects of the lab's involvement in the Network. In addition, a single Foundation staff person—the VBN Administrator—handled overall VBN logistics and administration and orchestrated the work of the individual lab's administrators.

### Partnering and the Power of Leveraging

To be successful, a network must actively engage the broader field in which it works, openly sharing the results of its efforts and incorporating advances achieved by others. In this way, network resources are highly leveraged, magnifying the impact of the network's funding, talent, technical capacities, and discoveries.

### Funders' Opportunity

A funding agency can accomplish much with modest resources. However, it must choose its targets carefully, articulate ambitious but measurable goals, recognize the importance of timing, be strategic, be trusting of its investigators and the dynamic of science, and be willing to experiment with new funding mechanisms. As a function of their independence, foundations have a particular opportunity to take on important but neglected societal issues and to try flexible new strategies for attacking them.

## Summary

The VBN more than met the objectives of The MacArthur Foundation, and it validated the field-development concepts that motivated its creation. Through the separate and collaborative research and training activities of Network laboratories, the two-week summer course, and an ongoing set of strategic planning and convening activities, the VBN (i) led in the development of proof-of-concept demonstrations of genetic methods for interrupting the transmission of selected parasitic and viral VBDs; (ii) developed and applied the first methods for producing transgenic mosquitoes; (iii) helped to create the rationale, strategy, and collaborative approach for sequencing the genomes of the most significant mosquito vectors of human malaria and dengue; (iv) produced novel understanding concerning the functions and operation of the insect immune system, illuminated molecular events and vulnerabilities during blood feeding and digestion and the molecular basis of olfaction in host-seeking; (v) developed and offered the BDV course and published the landmark textbook *The Biology of Disease Vectors*; and (vi) trained more than 600 students, postdoctoral researchers, and faculty from around the world, many of whom now fill leadership positions in vector biology or medical entomology; and (vii) recruited outstanding scientists from other fields to vector biology, many of whom transformed their entire laboratories to work on vectors and have become leaders in the field.

In conjunction with or following the VBN initiative, other organizations initiated or emphasized programs to target vectors for disease control using modern molecular approaches. WHO-TDR initiated a new program on parasite vector biology emphasizing modern genetic and population genetic research and training. The portfolio of vector grants exploded at the NIH, culminating in a Vector Biology study section, and Gates Grand Challenge grants and other foundation initiatives are targeting vectors for disease control using modern approaches. The WHO-TDR–funded effort to incorporate scientists from the developing world and disease-endemic countries into Network activities has greatly enriched the field, benefiting scientists from both North and South, and has yielded extraordinary collaborative studies and efforts to control VBDs. As a result, there is now a vibrant and productive community of basic and applied researchers, funding agencies, and interventionists working in what was at the time of the Network's creation a seriously neglected area of public health.

The output of the VBN in trained people, knowledge, tools, and approaches represents important additions to the public health armamentarium for control of VBDs. Indeed, new vector control capacity combined with vaccination and therapeutic programs provide a new exciting and integrated opportunity to control and perhaps regionally eradicate some of the VBDs that are such important causes of morbidity, mortality, and misery in humankind.

## Supporting Information

Figure S1 Participants of the Tucson, Arizona, meeting “Prospects for Malaria Control by Genetic Manipulation of its Vectors,” held January 27–31, 1991, sponsored by The MacArthur Foundation, World Health Organization Special Programme for Research and Training in Tropical Diseases, and the University of Arizona. Scientists with expertise in basic molecular biology, genetics, epidemiology, entomology, vector control, and public health met to discuss the use and role of molecular techniques in vector and disease control. The official report of the meeting was published by WHO-TDR (TDR/BCV/MAL-ENT/91).
(6.37 MB TIF)
