# Supplementary figures and images for: From Tucson to Genomics and Transgenics: The Vector Biology Network and the Emergence of Modern Vector Biology
Source: PLoS Negl Trop Dis. 2009 Mar 31;3(3):e343. doi: 10.1371/journal.pntd.0000343 (PMC2659576; doi:10.1371/journal.pntd.0000343)

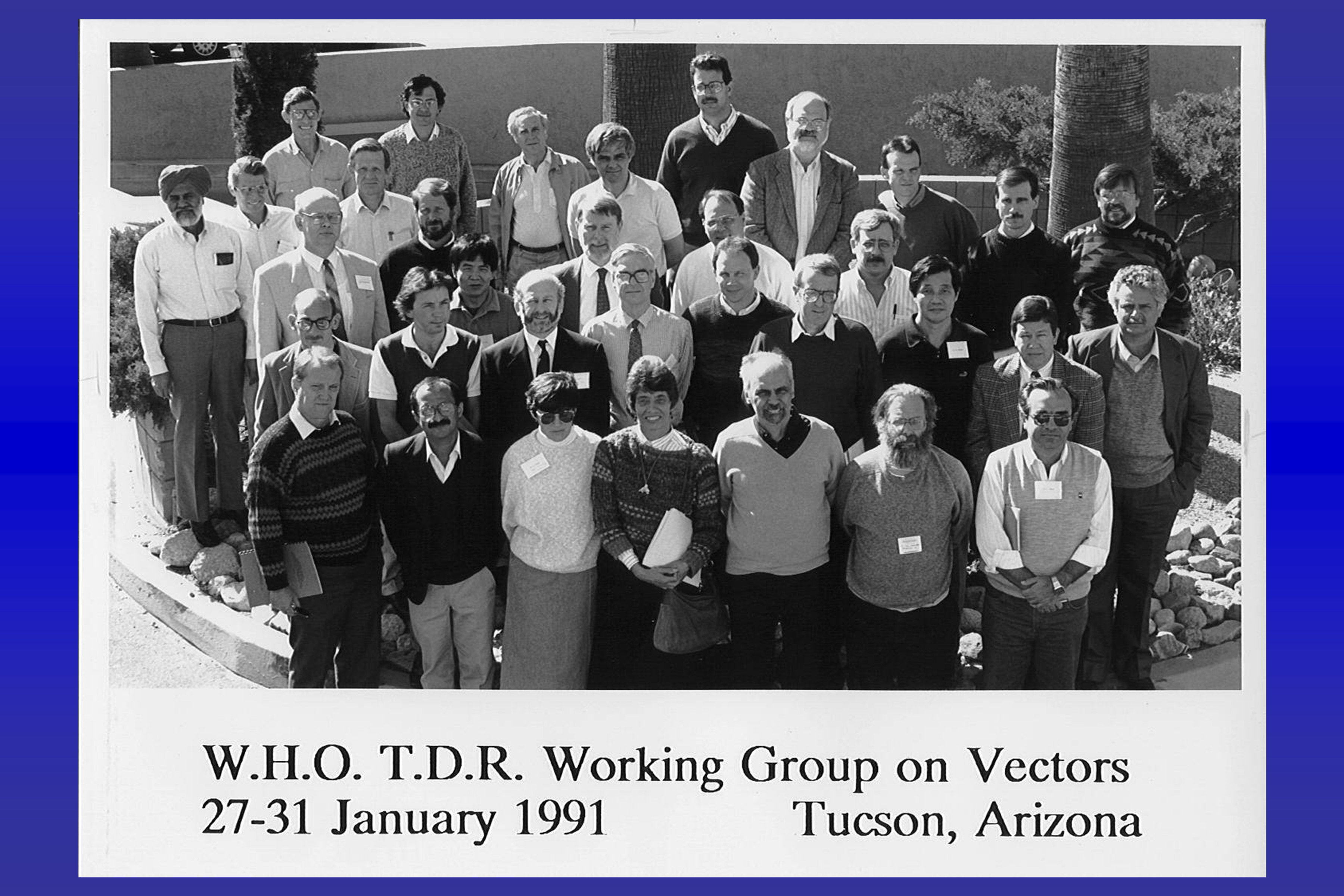

Supplement: Figure S1 — Participants of the Tucson, Arizona, meeting “Prospects for Malaria Control by Genetic Manipulation of its Vectors,” held January 27–31, 1991, sponsored by The MacArthur Foundation, World Health Organization Special Programme for Research and Training in Tropical Diseases, and the University of Arizona. Scientists with expertise in basic molecular biology, genetics, epidemiology, entomology, vector control, and public health met to discuss the use and role of molecular techniques in vector and disease control. The official report of the meeting was published by WHO-TDR (TDR/BCV/MAL-ENT/91). (6.37 MB TIF) [file pntd.0000343.s001.tif]
